# Commentary on: BreastGAN: Artificial Intelligence-Enabled Breast Augmentation Simulation

**DOI:** 10.1093/asjof/ojac028

**Published:** 2022-04-14

**Authors:** Jacob G Unger, Dayna Goltz

**Affiliations:** Dr Unger is a plastic surgeon in private practice, Nashville, TN, USA; and a breast surgery section contributing editor for ASJ Open Forum. Ms Goltz is an intern, Vanderbilt University, Nashville, TN, USA

See the Original Article here.

“BreastGAN: Artificial Intelligence-Enabled Breast Augmentation Simulation” written by Dr Chartier et al focuses on cutting-edge breast augmentation technology.^1^ I did some early research 15-20 years ago in three-dimensional (3D) imaging—in which laser scanning and reverse engineering computer-aided design (CAD) software were utilized to build models for breast augmentation. This article describes a significant step forward in terms of both computing power and ease of technological use, while also using very straightforward technology we are all familiar with. It uses standardized photography, possibly even photography taken with a smartphone. It then uses a neural network—without requiring significant manual work after the dataset is created—to help generate a possible future surgical result. The authors should be commended for their contributions regarding our ability to utilize the technology at our disposal to help create novel ways of providing patients with a better understanding of possible postoperative outcomes (Video).

While the article is a great first step, there are certainly a broad variety of limitations to utilizing this technology in the clinical arena at the moment. Importantly, all the photographs used in the article and for the dataset to help create postoperative images were from only one surgeon and did not have information based on implant sizing. Furthermore, all the images are only frontal images. Patients are often very interested in postoperative results from many different viewpoints, including lateral, oblique, three-quarters, and even cephalic and caudal views. The current legacy 3D systems can help surgeons create these images for patients, albeit with more work and at a higher cost to utilize.

This dataset, again, cannot be generalized as it is not used with other surgeons’ data and does not have any indication of implant sizing for the neural network to learn from. This is well documented and noted within the article. Perhaps with a large amount of future data, BreastGAN (Montreal, Canada) can be utilized for a more meaningful clinical application, which I think would be the future of this type of technology. The authors do indicate that it would require a very large body of photographic data from many different surgeons to create a more consistent and reliable simulated outcome. Hence, the data do not allow for any surgeon to use the images as they currently are. It would require large amounts of data, which—unlike facial recognition software that can pull billions of facial images from the internet—requires patient consent to use their preoperative and postoperative images. In my opinion, it requires not only data corresponding to each image on implant sizing—as discussed by the authors—but also data on BMI, chest wall diameter, tissue quality, tissue thickness, stretch, state of childbearing, age, etc.

The authors should certainly be commended for helping to open the door to exploring this novel space, which is likely the future of breast augmentation. However, we are not quite at the point where the data and computation are powerful enough to use the technology in a meaningful way to show possible simulated before and after images. I think that the images created by their data, which are shown at the end of the paper, are fairly impressive and represent realistic breast shapes overall. However, if the images are carefully parsed, they do not display very accurate breast size, shape, and positioning on the chest wall when comparing the simulated result with the actual final outcomes. That, of course, is due to the limited variability of training data due to the use of only one surgeon’s experience.

I look forward to commentaries and future papers based on this concept. Furthermore, I look forward to future research addressing the open-ended question of artificial intelligence-enabled software understanding a specific surgeon’s approach. 

## References

[1] Chartier C, Watt A, Chandawarkar A, et al BreastGAN: artificial intelligence-enabled breast augmentation simulation. Aesthet Surg J Open Forum. 2022;4:1-6. doi: 10.1093/asjof/ojab052 PMC8781773 35072073

